# Clinical Effectiveness of Different Technologies for Diabetes in Pregnancy: Systematic Literature Review

**DOI:** 10.2196/24982

**Published:** 2021-04-28

**Authors:** Claudia Eberle, Maxine Loehnert, Stefanie Stichling

**Affiliations:** 1 Medicine with Specialization in Internal Medicine and General Medicine Hochschule Fulda - University of Applied Sciences Fulda Germany

**Keywords:** diabetes technologies, diabetes management, pregnancy, digital health, eHealth, systematic review

## Abstract

**Background:**

Hyperglycemia in pregnancy occurs worldwide and is closely associated with health issues in women and their offspring, such as pregnancy and birth complications, respectively, as well as comorbidities, such as metabolic and cardiovascular diseases. To optimize the management of diabetic pregnancies, sustainable strategies are urgently needed. Investigation of constantly evolving technologies for diabetes that help to manage pregnancy and health is required.

**Objective:**

We aimed to conduct a systematic review to assess the clinical effectiveness of technologies for diabetes in pregnancy.

**Methods:**

Relevant databases including MEDLINE (PubMed), Cochrane Library, Embase, CINAHL, and Web of Science Core Collection were searched in September 2020 for clinical studies (2008-2020). Findings were organized by type of diabetes, type of technology, and outcomes (glycemic control, pregnancy- and birth-related outcomes, and neonatal outcomes). Study quality was assessed using Effective Public Health Practice Project criteria.

**Results:**

We identified 15 randomized controlled trials, 3 randomized crossover trials, 2 cohort studies, and 2 controlled clinical trials. Overall, 9 studies focused on type 1 diabetes, 0 studies focused on gestational diabetes, and 3 studies focused on both type 1 diabetes and type 2 diabetes. We found that 9 studies were strong quality, 11 were moderate quality, and 2 were weak quality. Technologies for diabetes seemed to have particularly positive effects on glycemic control in all types of diabetes, shown by some strong and moderate quality studies. Positive trends in pregnancy-related, birth-related, and neonatal outcomes were observed.

**Conclusions:**

Technologies have the potential to effectively improve the management of diabetes during pregnancy. Further research on the clinical effectiveness of these technologies is needed, especially in pregnant women with type 2 diabetes.

## Introduction

Hyperglycemia in pregnancy occurs worldwide and is closely associated with short- and long-term health issues, such as pregnancy complications in women and birth complications in offspring, and comorbidities, such as type 2 diabetes, as well as metabolic and cardiovascular diseases [1]. Approximately 20.4 million live births (16%) suffered from some form of hyperglycemia in pregnancy in 2019, worldwide [1]—84% were defined as gestational diabetes, 7.9% were diagnosed prior to pregnancy, and 8.5% were type 1 or type 2 diabetes first observed in pregnancy [1].

In 2019, 1,566,993.4 live births in North America and the Caribbean; 2,001,816.8 live births in Europe; and 6,594,159.4 live births in southeast Asia were affected by hyperglycemia in pregnancy [1]. Interestingly, the prevalence of gestational diabetes is rising quickly and was estimated to be 20.8% in North America and the Caribbean, 16.3% in Europe, and 27.0% in southeast Asia in 2019 [1]. The number of unreported cases is assumed to be high.

Hyperglycemia in pregnancy is associated with adverse pregnancy- and birth-related outcomes for mothers and offspring, such as increased risk of preeclampsia, cesarean deliveries, macrosomia, and shoulder dystocia [2-6], but hyperglycemia in pregnancy is also associated with adverse long-term consequences in mothers and children, such as subsequent increased risk of type 2 diabetes, obesity, metabolic syndrome, cardiovascular diseases, or even depression [2,4,5,7,8]. Based on the hypotheses of perinatal programming (fetal programming, developmental programming, transgenerational programming), intrauterine exposure to hyperglycemia is clearly associated with obesity, glucose, intolerance, type 2 diabetes, insulin resistance, metabolic syndrome, high blood pressure, and cardiovascular diseases in postnatal life of the offspring [5,9,10].

Sustainable strategies are urgently needed to effectively improve the management of diabetes in pregnancy with seamlessly integrated technological support for hyperglycemia in pregnancy. Technologies for diabetes, including hardware, devices, and software, are constantly evolving and can help to manage the condition, improving lives and health [11]. *Care 4.0* is a new paradigm to develop digital health and care services by focusing on trusted integrated networks of organizations, people, and technologies [12].

Recently, we demonstrated first insights in clinical effectiveness and successful approaches of mobile health (mHealth) apps as well as telemetric implementations to improve diabetic management in pregnancy [13,14]. Diabetes-specific mHealth apps and telemetric approaches improved clinical outcomes in the management of gestational diabetes effectively.

As a matter of course, there are many more technologies that have proven to be effective in diabetes management in general, for example, continuous glucose monitoring systems [15,16], continuous subcutaneous insulin infusions [17,18], and sensor augmented insulin pumps or closed-loop systems [19,20].

A previous review [21] examined the capability of diabetes technologies in the treatment of diabetic pregnancies; however, to further optimize management of diabetes in pregnancy, up-to-date analyses of technology for diabetes in pregnancies are needed. Therefore, we conducted a systematic review to assess the clinical effectiveness of different technologies for diabetes in pregnancies by analyzing glycemic control, pregnancy- and birth related outcomes, and neonatal outcomes.

## Methods

### Data Sources and Searches

A systematic review was carried out using PRISMA guidelines [22]. MEDLINE (PubMed), Cochrane Library, Embase, CINAHL, and Web of Science Core Collection were systematically searched for studies published from January 2008 until September 2020 (Multimedia Appendix 1). The following keywords were used as title and abstract search terms and selected from Medical Subject Headings and Embase subject headings databases: “diabetes mellitus,” “pregnancy,” “gestational diabetes mellitus,” “mobile application,” “insulin pump,” “continuous glucose monitoring,” “CGM,” “flash glucose monitoring,” “FGM,” “insulin infusion system” (Multimedia Appendix 2). Using a priori criteria for eligibility, 2 reviewers screened and selected the studies independently. We screened titles and abstracts, assessed full texts, and manually searched reference lists and Google Scholar.

### Eligibility Criteria

We included peer-reviewed studies, published in German and English, that investigated the clinical effectiveness of diabetes technologies in pregnancies affected by hyperglycemia, including pre- and postconception. Studies with pregnant woman with a diagnosis of gestational diabetes or with preexisting type 1 diabetes or type 2 diabetes and that clearly reported the type of diabetes investigated were included. We included studies examining gestational diabetes patients only, type 1 diabetes only, type 2 diabetes only, and studies with mixed pregestational diabetes (type 1 diabetes and type 2 diabetes) groups. We included prospective controlled trials (clinical and observational trials). The following types of technologies were included: continuous glucose monitoring systems, continuous subcutaneous insulin infusions, sensor augmented insulin pumps, closed-loop systems, and mHealth apps. We involved studies examining glycemic control, pregnancy- and birth-related, and neonatal outcomes. Poster, comments, letter, study protocols, notes, and proceedings papers were excluded.

### Data Extraction and Synthesis

We extracted the most common glycemic control (eg, hemoglobin A_1c_ [HbA_1c_], fasting blood glucose, hyper- or hypoglycemia, insulin dose), pregnancy- and birth-related (eg, preterm birth, gestational weight gain), and neonatal (eg, birthweight, macrosomia) outcomes as well as author, year, study design, type of diabetes, type of technology, sample size, and main results. Findings were organized by type of diabetes, type of technology, and outcomes.

### Quality Assessment

We applied Effective Public Health Practice Project (EPHPP), a validated tool that is suitable for intervention studies such as randomized controlled trials and controlled clinical trials. EPHPP is composed of criteria regarding selection bias, study design, confounders, blinding, data collection methods, withdrawals, and dropout. Study quality is rated as strong (SQ), moderate (MQ), or weak (WQ) [23].

## Results

### Study Selection and Characteristics

The search yielded 974 records, of which 754 remained after removing duplicates (3 records were identified through reference lists and Google Scholar). Of those, 693 were excluded based on titles and abstracts with our inclusion and exclusion criteria, and the full texts of 61 remaining publications were screened. Finally, 22 studies were included in this systematic review: 15 randomized controlled trials, 3 randomized crossover trials, 2 cohort studies, and 2 controlled clinical trials. Overall, 9 studies focused on type 1 diabetes, 10 studies focused on gestational diabetes, and 3 studies focused on type 1 diabetes and type 2 diabetes (mixed populations). We rated 9 studies as strong quality, 11 studies as moderate quality, and 2 studies as weak quality (Multimedia Appendix 3). Table 1 gives an overview of the technologies, outcomes, and number of study participants..

**Table 1:** Overview of the technologies, outcomes, and study participants.

Population, technology, and outcome			Studies, n	Intervention group, n	Control group, n
**Type 1 diabetes**					
	**Continuous glucose monitoring systems**				
		HbA_1c_^a^	3	199	236
		Maternal hypoglycemia	2	172	177
		Insulin dose and insulin requirements	3	199	236
		Cesarean and vaginal deliveries	3	139	174
		Preterm birth^b^	3	139	174
		Maternal weight gain	2	112	115
		Neonatal birthweight	2	127	159
		Neonatal macrosomia	2	112	113
		Large for gestational age	2	127	113
	**Continuous subcutaneous insulin infusion**				
		HbA_1c_	4	218	485
		Insulin dose	2	145	292
		Maternal hypoglycemia	2	160	237
		Maternal weight gain	2	223	273
		Cesarean delivery	3	271	353
		Preterm birth	3	271	353
		Maternal hypertension and preeclampsia	2	159	196
		Neonatal birthweight	2	158	195
		Macrosomia (≥4000 g)	2	158	195
		Large for gestational age	2	158	195
		Small for gestational age	2	158	195
		Admission to a higher level of neonatal care	2	158	195
		Neonatal hypoglycemia	2	158	195
	**Closed-loop systems**				
		Hypoglycemic events, insulin dose	2	16	16
**Gestational diabetes**					
	**Continuous glucose monitoring systems**				
		HbA_1c_	3	96	99
		Fasting blood glucose	2	85	87
		Preterm birth	3	96	99
		Cesarean and vaginal deliveries	3	87	92
		Maternal weight gain	2	76	80
		Neonatal birthweight	4	147	154
		Macrosomia (≥4000 g)	4	147	154
		Large for gestational age	3	87	92
		Small for gestational age	3	87	92
		Admission to a higher level of neonatal care	3	96	99
		Neonatal hypoglycemia	3	136	142
	**mHealth apps**				
		HbA_1c_	2	164	161
		Fasting blood glucose	2	69	62
		Off-target fasting blood glucose measurement	2	124	120
		Patient compliance^c^	2	124	120
		Pregnancy-induced hypertension or preeclampsia	3	218	212
		Preterm birth	2	158	152
		Vaginal and cesarean deliveries	5	390	393
		Neonatal birthweight	3	218	211
		Macrosomia	3	233	231
		Neonatal hypoglycemia	4	277	263
		Admission to a higher level of neonatal care	4	330	330
**Type 1 and type 2 diabetes**					
	**Continuous glucose monitoring systems**				
		HbA_1c_	2	117	108
		Plasma glucose	1	79	75
		Preeclampsia	2	117	108
		Cesarean delivery	2	117	108
		Preterm birth	2	117	108
		Neonatal birthweight	2	117	108
		Large for gestational age	2	117	108
		Neonatal hypoglycemia	2	117	108
	**Continuous subcutaneous insulin infusion**				
		HbA_1c_	1	24	18

^a^HbA_1c_: hemoglobin A_1c_.

^b^*Preterm delivery* was defined as <37 weeks.

^c^*Patient compliance* was defined as ratio between actual and instructed blood glucose measurements×100.

### Type 1 Diabetes: Overview

Of 9 publications analyzing 379 women in intervention (IG) and 763 in control groups (CG), 3 studies examined continuous glucose monitoring system, 4 studies examined continuous subcutaneous insulin infusion, and 2 studies examined closed-loop systems.

### Type 1 Diabetes: Continuous Glucose Monitoring Systems

In general, 3 randomized controlled trials [24-26], analyzing 199 women in intervention groups and 236 women in control groups, were identified.

#### Glycemic Control Outcomes

HbA_1c_ values declined in all interventions. Feig et al [25] (MQ) observed significantly lower HbA_1c_ values in the intervention group than the control group (mean difference −0.19%; 95% CI −0.34 to −0.03; *P*=.0207). Cordua et al [24] (SQ) found positive but nonsignificant effects (IG: 6.0% vs CG: 6.2%; *P*=.23), and Petrovski et al [26] (SQ) reported significantly improved HbA_1c_ values (*P*<.05) in both groups (real-time continuous glucose monitoring system and intermittent continuous glucose monitoring system).

Petrovski et al [26] (SQ) found significantly less severe events of maternal hypoglycemia (real-time continuous glucose monitoring system group: *P*<.05), whereas Feig et al [25] (MQ) found time spent hypoglycemic was comparable in both groups (3% vs 4%; *P*=.10).

The intervention groups tended to have better target insulin dose or requirement values than those of the control groups (*P*=.14 [25], MQ; *P*=.08 [24], SQ). Petrovski et al [23] (SQ) did not find significant differences between constant and intermittent continuous glucose monitoring system (*P*>.05).

#### Pregnancy- and Birth-Related Outcomes

Cesarean delivery rates were clearly lower in the intervention groups (MQ, *P*=.18 [25]; SQ, *P*<.05 [23]). Cordua et al [24] (SQ) found significantly more vaginal deliveries in the intervention groups (IG: 74%; CG: 54%; *P*=.08).

All studies found slightly fewer preterm births in the interventions groups (Feig et al [25] <37 weeks: *P*=.57; <34 weeks: *P*=.19; Petrovski et al [23]: *P*>.05, Cordua et al [24]: *P*=.84).

Maternal gestational weight gains were lower in the continuous glucose monitoring system group (Feig et al [25] +8.9 kg vs +9.7 kg, *P*=.09) and the intermittent continuous glucose monitoring system group (Petrovski et al [26] +12.9 kg vs 13.4 kg, *P*>.05).

#### Neonatal Outcomes

Feig et al [25] (MQ) displayed a slightly lower mean birthweight in the continuous glucose monitoring system group (*P*=.37), whereas Cordua et al [24] (SQ) found a lower mean birthweight in their control group (*P*=.19).

Macrosomia rates were lower in intervention (Feig et al [25], MQ, IG: 23%; CG: 27%; *P*=.62) and real-time continuous glucose monitoring system (Petrovski et al [26], SQ, *P*>.05) groups.

Feig et al [25] found rates for large for gestational age were significantly lower in the intervention groups (*P*=.021), whereas Cordua et al [24] found no significant differences (*P*=.08).

### Type 1 Diabetes: Continuous Subcutaneous Insulin Infusion

In general, 2 cohort studies [27,28], 1 randomized controlled trials [29], 1 randomized crossover trial [30] analyzing 148 intervention and 495 control participants were identified.

#### Glycemic Control Outcomes

Overall, 3 studies found positive effects on HbA_1c_ levels in favor of continuous subcutaneous insulin infusion. Jotic et al [28] (SQ) found a significant lower mean HbA_1c_ level in their intervention group than that in the control group at follow-up (6.5% intervention vs 6.8% control, *P*=.002). Cyganek et al [27] (SQ) also reported significant improvements in HbA_1c_ within all their treatment groups but did not find significant differences between groups.

Gutaj et al [30] (SQ) found significant lower insulin doses in the intervention group (0.54 U/kg/day intervention vs 0.63 U/kg/day control, *P*=.02), whereas Cyganek et al [27] (SQ) showed significantly lower daily insulin doses within each treatment group but no significant differences between groups.

Jotic et al [28] (SQ) reported that in early pregnancy, the majority of women on continuous subcutaneous insulin infusion had significantly fewer instances of hypoglycemia (*P*<.01), but Cyganek et al [27] (SQ) found fewer instances of severe hypoglycemia in their control group (*P*=.04).

#### Pregnancy- and Birth-Related Outcomes

Cyganek et al [27] (SQ) found less weight gain (+13.0 kg) in women in the control group receiving multiple daily injections than in the other groups (+14.7 kg for those only continuous subcutaneous insulin infusion; +15.2 kg for those moving from multiple daily injections to continuous subcutaneous insulin infusion; *P*=.005), and Feig at al [29] (MQ) did not find significant differences between groups (IG: mean +13.5 kg, SD 5.4 kg; CG: mean+13.5 kg, SD 0.7 kg; *P*=.48).

The studies could not find any clearly positive effects regarding cesarean deliveries in favor of continuous subcutaneous insulin infusion. Cyganek et al [27] (SQ) reported that the proportion of cesarean deliveries was larger in women using insulin pumps (no *P* value reported) because of clinical factors such as age, duration of diabetes, and microvascular complications. Feig et al [29] (MQ) (IG: n=81 vs CG: n=74, *P*=.32) and Jotic et al [28] (SQ) (IG: n=12 vs CG: n=13, *P*=.20) found no significant differences between groups.

There were no significant differences in preterm births between groups (*P*=.30 [27]; pump: n=39, 35%, vs multiple daily injections: n=35, 30%, *P*=.10 [29]; continuous subcutaneous insulin infusion n=13, 27.7%, vs multiple daily injections n=13, 16.3%, *P*=.17 [28]).

Feig et al [29] (MQ) found significantly fewer women with hypertension in the control group (IG: 14.4% vs CG: 5.2%; *P*=.025), whereas Jotic et al [28] (SQ) reported a lower, but not significantly so, rate of women with hypertension in their intervention group (*P*=.09). Feig et al [26] (MQ) also found fewer women with preeclampsia in the control group (*P*=.31), whereas Jotic et al [28] (MQ) noted fewer women with preeclampsia in the intervention group (IG: n=1, CG: n=3, *P* value not available).

#### Neonatal Outcomes

Neonatal birthweight was marginally higher in intervention groups (Feig et al [29], MQ, *P*=.91; Jotic et al [28], SQ, *P*=.98).

Jotic et al [28] (SQ) found fewer instances of macrosomia in the intervention group (*P*=.46), while Feig et al [29] (MQ) found slightly more instances in the intervention group (*P*=.89).

Jotic et al [28] reported fewer instances of newborns being large for gestational age in the intervention group (*P*=.59 [28]), whereas Feig et al found slightly more newborns who were large for gestational age in the interventions group (*P*=.55).

Jotic et al [28] (SQ) found no instances of newborns being small for gestational age in the intervention and 5 (4%) instances of newborns being small for gestational age in the control group (*P* value not available), whereas Feig et al [29] (MQ) observed 3 and 1 instances of newborns being small for gestational age in the intervention and control groups, respectively (*P* value not available).

Feig et al [29] (MQ) and Jotic et al [28] (SQ) found differences in admissions to higher level neonatal care in favor of the control groups (IG: 44.5% vs CG: 29.6%, *P*=.02 [29]; and *P*=.28 [28]).

Jotic et al [28] (SQ) found slightly fewer instances of neonatal hypoglycemia in the intervention group (IG: n=5, CG: n=6, *P*=.54), whereas Feig et al [29] (MQ) reported a significant difference in favor of the control group (IG: 31.8% vs CG: 19.1%, *P*=.05).

### Type 1 Diabetes: Closed-Loop Systems

Closed-loop systems were investigated in 2 randomized crossover trials including a total of 32 women comparing closed-loop systems with sensor augmented insulin pumps [29,31].

Stewart et al [31] found a significant difference between groups in percentage of time that blood glucose levels were between 63 and 140 mg/dL for overnight evaluation (*P*=.002) as well as day and night evaluation (*P*<.001) in favor of the closed-loop group [31], while Stewart et al [29] (MQ) found no significant difference (closed-loop: 62.3% vs sensor augmented insulin pumps: 60.1%, 95% CI 24.1% to 8.3%, *P*=.47).

Stewart et al [32] (MQ) reported significant fewer hypoglycemic events during the closed-loop treatment (n=8 vs n=12.5, *P*=.04) [32], whereas Stewart et al [31] (MQ) found no significant differences in overnight analysis (closed-loop: median 3.0; sensor augmented insulin pumps: median 2.5, *P*=.68) or day and night analysis (closed-loop: median 11.0, sensor augmented insulin pumps: median 12.0, *P*=.19).

Neither Stewart et al [32] (MQ) (closed-loop 43.7 vs sensor augmented insulin pumps 41.5 units/day, *P*=.56) nor Stewart et al [31] (MQ) (closed-loop 58.2 vs sensor augmented insulin pumps 59.8 units/day, *P*=.56) found significant differences between treatments regarding insulin dose [31,32].

### Gestational Diabetes: Overview

In general, 4 studies focused on continuous glucose monitoring system, and 6 studies focused on mHealth apps (overall: IG: n=555; CG: n=609).

### Gestational Diabetes: Continuous Glucose Monitoring Systems

We identified 4 randomized controlled trials (IG: n=147, CG: n=154). Whereas 3 studies used self-monitoring blood glucose as control [33-35], 1 study used blinded continuous glucose monitoring system which did not display the glucose readings to the participant [36].

#### Glycemic Control Outcomes

All studies reported clearly lower HbA_1c_ values in their intervention groups. Paramasivam et al [34] (MQ) found a significant difference in favor of the intervention group (mean 5.2%, SD 0.4% vs mean 5.6%, SD 0.6%; *P*=.006). Alfadhli et al [33] (*P*=.168) (MQ) and Lane et al [36] (*P*=.3) (SQ) found lower mean HbA_1c_ values in their intervention groups (*P*>.05).

Paramasivam et al [34] (MQ) (*P*=.101) and Alfadhli et al [33] (*P*=.092) (MQ) found lower fasting blood glucose values in the intervention groups.

#### Pregnancy- and Birth-Related Outcomes

Alfadhli et al [33] (MQ) (IG=16.3%, CG=9.5%, *P*=.373), Paramasivam et al [34] (MQ) (IG: n=3, 12%; CG: n=1, 4%; *P*=.609) and Lane et al [36] (SQ) (IG: n=1, 9.1%; CG: n=2, 16.7%; *P*>.999) found no significant differences in the occurrence of preterm births.

Paramasivam et al [34] (MQ) reported clearly more vaginal deliveries in the intervention group (*P*=.258) [34], while Wei et al [35] (MQ) (*P*=.370) and Lane et al [36] (SQ) (*P*>.999) noted slightly more cesarean deliveries in the control groups.

Both studies showed positive effects on maternal weight gain. Wei et al [35] (MQ) reported that the intervention participants had significantly more appropriate and less excessive weight gain (*P*=.039) and Paramasivam et al [34] (MQ) mentioned slightly less weight gain in the intervention group (*P*=.917).

#### Neonatal Outcomes

Wei et al [35] (*P*=.084), Alfadhli et al [33] (MQ) (*P*=.130) and Paramasivam et al [34] (MQ) (*P*=.311) reported a lower, yet not significant, mean birthweight of newborns in the intervention groups. Lane et al [36] (SQ) reported a higher mean birthweight of newborns in the intervention group (*P*=.4).

Wei at al [35] (MQ) (*P*=.410) and Alfadhli et al [33] (MQ) (*P*=.488) reported fewer instances of macrosomia in the intervention groups, while Lane et al [36] (SQ) noted more instances of macrosomia in the intervention group (*P*=.2), and Paramasivam et al (MQ) [34] found none.

Wei et al [35] (MQ) (*P*=.071) and Paramasivam et al [34] (MQ) (*P*=.490) found clearly fewer instances of newborns being large for gestational age in their intervention groups and, in contrast, Lane et al [36] (SQ) found more instances of newborns being large for gestational age in the intervention group (*P*=.2).

Lane et al [36] (SQ) (*P>*.999) and Wei et al [35] (MQ) (*P>*.999) reported very slightly fewer instances of newborns being small for gestational age in the intervention groups. Paramasivam et al [34] reported none.

Alfadhli et al [33] (MQ) (*P*=.653) reported slightly more admissions, Lane et al [36] (*P*>.999) noted slightly fewer, and Paramasivam et al [34] (MQ) (both groups n=1, 4%) observed the same number of admissions (*P*>.999) to a higher level of neonatal care.

Wei at al [35] (MQ) (*P*=.410) and Paramasivam et al [34] (MQ) (*P*>.999) reported slightly fewer instances of neonatal hypoglycemia among intervention participants whereas Alfadhli et al [33] (MQ) (*P*=.758) found slightly fewer instances of neonatal hypoglycemia in the control group.

### Gestational Diabetes: mHealth Apps

We identified 5 randomized controlled trials and 1 controlled clinical trial investigating a total of 408 women in the intervention and 405 women in the control groups.

#### Glycemic Control Outcomes

Overall, the intervention groups showed lower HbA_1c_ values than the control groups. Guo et al [37] (SQ) displayed a significant difference in favor of the intervention group (–1.3% intervention vs –0.6% control, *P*<.001), while Mackillop et al [38] (WQ) recognized a slight increase in both groups (IG: 0.02% per 28 days; CG: 0.03% per 28 days; 95% CI –0.05 to 0.03).

Both Yang et al [39] (MQ) (*P*<.001) and Bromuri et al [40] (MQ) (*P*<.001) found clear, significant differences in fasting blood glucose levels favoring the intervention groups.

Miremberg et al [41] (SQ) (*P*<.001) as well as Guo et al [37] (SQ) (*P*<.001) found significant differences in off-target fasting blood glucose measurement favoring the intervention groups [37,41].

Guo et al [37] (SQ) (*P*<.001) and Miremberg et al [41] (SQ) (*P*<.001) reported significant differences in support of the intervention groups in patient compliance (defined as ratio between actual and instructed blood glucose measurements×100).

#### Pregnancy- and Birth-Related Outcomes

All intervention groups showed lower rates of pregnancy-induced hypertension or preeclampsia, but no significant differences were found by Mackillop et al [38] (WQ) (*P*=.22), Miremberg et al [41] (SQ) (*P*>.99), or Yang et al [39] MQ) (*P*=.347).

Overall, there were fewer preterm births in the intervention groups, but neither Yang et al [39] (MQ) (*P*=.248) nor Mackillop et al [38] (WQ) (*P*>.05) found significant differences.

Positive intervention effects—more vaginal deliveries and less cesarean sections—were observed in almost all studies [37-39,41,42]; 2 studies showed statistical significance (Borgen et al [42], WQ, *P*=.03; Mackillop et al [38], WQ, *P*=.005).

#### Neonatal Outcomes

The results show a clear trend toward lower birthweight in the newborns of the intervention groups, but no significant differences were found by Mackillop et al [38] (WQ) (*P*=.18), Miremberg et al [41] (SQ) (*P*=.878), or Yang et al [39] (MQ) (*P*=.988).

Fewer instances of macrosomia occurred in the intervention groups than in the control groups, but no significant differences were found by Guo et al [37] (SQ) (*P*=.295), Yang et al (MQ) [39] (*P*=.542) and Borgen et al [42] (WQ) (*P*=.69).

In general, most studies showed positive intervention effects. Guo et al [37] (SQ)(*P*=.185), Mackillop et al (WQ) [38] (*P*=.42) and Yang et al (MQ)[39] (*P* value not available) observed slightly fewer instances of neonatal hypoglycemia for intervention participants.

The results show a trend toward infants of mothers in the interventions groups being transferred less often to neonatal intensive care units (Borgen et al [42], WQ, *P*=.38; Mackillop et al [38], WQ, *P*=.08; Miremberg et al [41], SQ, *P*>.99; Yang et al [39], MQ, *P*=.657).

### Type 1 and Type 2 Diabetes: Overview

In total, 3 studies analyzing 141 women in the intervention and 126 women in the control groups were included [43-45]. Two studies observed continuous glucose monitoring system and one insulin pumps.

### Type 1 and Type 2 Diabetes: Continuous Glucose Monitoring Systems

Overall, 2 randomized controlled trials analyzed 117 intervention and 108 control participants. Secher et al [45] included 123 women with type 1 diabetes and 31 women with type 2 diabetes, while Murphy et al [44] included 45 women with type 1 diabetes and 25 type 2 diabetes.

#### Glycemic Control Outcomes

Murphy et al [44] (MQ) noted that HbA_1c_ levels were consistently and significantly lower in the intervention group (gestation 32-36 weeks: *P*=.007), while Secher et al [45] (MQ) reported comparable HbA_1c_ values (IG: 6.1%, 95% CI 5.1%-7.8%; CG: 6.1%, 95% CI 4.8%-8.2%; *P*=.39).

Median self-monitored plasma glucose levels at 33 weeks were comparable between groups (IG: 6.2 mmol/L, 4.7-7.9; CG: 6.2 mmol/L, 4.9-7.9) [45].

#### Pregnancy- and Birth-Related Outcomes

Secher et al (MQ) and Murphy et al (MQ) observed very slightly more instances of preeclampsia in the treatment groups (*P*=.83 [45] and *P*=.5 [44], respectively).

Secher et al [45] (MQ) observed a lower rate of cesarean delivery (*P*=.30) and Murphy et al [44] (MQ) reported a lower emergency cesarean delivery rate (*P*=.3) in the intervention group.

Murphy et al [44] (MQ) observed slightly fewer preterm births (*P*=.8), whereas Secher et al [45] (MQ) reported slightly more preterm births (*P*=.47) in the treatment group.

#### Neonatal Outcomes

Murphy et al [44] (MQ) found significantly lower median birthweight percentile in the intervention group (69 vs 93, *P*=.02), whereas Secher et al [45] (MQ) found the birthweight was slightly higher in the intervention group (3510 g vs 3436 g, *P*=.80).

Murphy et al [44] (MQ) reported that fewer newborns who were extremely large for gestational age (≥97.7th percentile) were born to intervention participants (14% vs 30%, *P*=.1 ]), whereas Secher et al [45] (MQ) reported slightly more newborns who were extremely large for gestational were born to participants in the treatment group (*P*=.19 [45]).

Both studies reported clearly fewer instances of neonatal hypoglycemia in the intervention groups (36% vs 40%, *P*=.62 [45]; and 8% vs 17%, *P*=.5 [44])

### Type 1 and Type 2 Diabetes: Continuous Subcutaneous Insulin Infusion

One study [43] examined continuous subcutaneous insulin infusion compared to conventional insulin therapy (intervention n=24 vs control n=18). Kernaghan et al [43] (SQ) included n=1 women with type 2 diabetes.

The intervention group showed lower mean HbA_1c_ levels in the first (*P*=.41) and second (*P*=.27) trimester. The birthweight *z* score was slightly lower in the control group (*P*=.86). The mean estimated fetal weight *z* score was lower in the intervention group (*P*=.16).

## Discussion

### Principal Results

In general, technologies seemed to have particularly positive effects on glycemic control in all types of diabetes, as shown by studies of strong and moderate quality. For type 1 diabetes, there were no studies evaluating the effectiveness of mHealth apps and, for gestational diabetes, there were no studies that evaluated the effectiveness of continuous subcutaneous insulin infusion or closed-loop systems. It is particularly useful for women with pregestational diabetes to use mHealth apps, as they can become familiar with them before pregnancy. Furthermore, there is a lack of research focusing on the effectiveness of technologies for pregnant women with type 2 diabetes.

### Type 1 Diabetes

#### Continuous Glucose Monitoring Systems

Overall, glycemic control improved with continuous glucose monitoring systems in strong and moderate quality studies, especially HbA_1c_. Positive trends were observed for hypoglycemia and insulin dose. Intermittent continuous glucose monitoring systems seemed to be preferable to real-time continuous glucose monitoring systems, with fewer cesarean deliveries and more vaginal deliveries; however, the findings were not statistically significant. Regarding maternal weight gain and preterm births, improvements seem to be possible through the use of continuous glucose monitoring system, especially intermittent continuous glucose monitoring systems. Birthweight, large for gestational age, macrosomia, and neonatal hypoglycemia rates showed trends for improvement with continuous glucose monitoring system, as some moderate and strong quality studies showed nonsignificant, positive effects.

#### Continuous Subcutaneous Insulin Infusion

Overall, continuous subcutaneous insulin infusion improved glycemic control, especially HbA_1c_ and insulin dose, as most strong and moderate quality studies showed positive intervention effects. In the case of maternal hypoglycemia, one study [28] found significantly fewer instances in the intervention group, but another study [27] found fewer instances in the control group.

No clear trend regarding the effectiveness of continuous subcutaneous insulin infusion can be derived regarding pregnancy- and birth-related outcomes such as maternal weight gain, preterm births, cesarean delivery, maternal hypertension, or preeclampsia. There were no clear trends in relation to neonatal outcomes such as birthweight, large for gestational age, small for gestational age, macrosomia, hypoglycemia, and admission to high-level neonatal care because too little data were available.

#### Closed-Loop Systems

A trend toward improved glycemic control was shown in moderate quality studies, but the sample size was very small (n=32 patients).

### Gestational Diabetes

#### Continuous Glucose Monitoring Systems

In general, continuous glucose monitoring system improved glycemic control, particularly HbA_1c_ and fasting blood glucose levels in strong and moderate quality studies; however, there was no obvious trend regarding 2-hour plasma blood glucose level and insulin dose. There were also no clear trends regarding preterm births or cesarean and vaginal deliveries. Both studies examining maternal weight gain showed positive but nonsignificant intervention effects.

Moreover, continuous glucose monitoring system improved birthweight, macrosomia, large for gestational age, small for gestational age, and neonatal hypoglycemia measures compared to those in control groups, though not significantly. Overall, there was no clear trend with respect to admission to higher level of neonatal care.

#### mHealth Apps

Gestational diabetes–specific mHealth apps displayed positive, significant effects in glycemic control outcomes such as HbA_1c_ level, fasting blood glucose level, off-target blood glucose measurement, and patient adherence (actual vs instructed blood glucose measurements) in strong and moderate quality studies. mHealth apps improved pregnancy-induced hypertension or preeclampsia and preterm birth outcomes, but not significantly. In addition, the treatments showed positive effects regarding vaginal deliveries and cesarean deliveries. Gestational diabetes–specific mHealth apps indicated noticeable positive, but not significant effects, on neonatal outcomes such as birthweight, macrosomia, hypoglycemia, and rate of admission to a higher level of care.

### Type 1 and Type 2 Diabetes

#### Continuous Glucose Monitoring Systems

The sample sizes of the studies were very small. One moderate-quality study [45] could not find any clear differences in HbA_1c_ values between intervention and control group, while another moderate-quality study [44] showed significant positive intervention effects. Cesarean deliveries were required less often. There was no obvious trend regarding preeclampsia and preterm births. While no clear effects were found regarding birthweight and being large for gestational age, continuous glucose monitoring system showed an improvement in terms of neonatal hypoglycemia outcomes, but not significantly.

#### Continuous Subcutaneous Insulin Infusion

One strong-quality study [43] indicated positive trends in terms of HbA_1c_, birthweight *z* score, and estimated fetal weight *z* score.

### Strengths and Limitations

To our knowledge, this is the first systematic review of different technologies for diabetes that differentiated between various types of diabetes. Hence, our review opens up new perspectives on the topic hyperglycemia in pregnancy. However, the body of research included in this review may be limited depending on the type of diabetes and technology. Further research, with larger sample sizes and that takes into account women in the pre-conception phase, is needed, especially. Furthermore, we included only German and English papers, and we performed a qualitative analysis.

### Comparison With Prior Work

Our results are in line with those from other reviews. In their systematic review, Feig et al [46] also reported that continuous glucose monitoring system improved HbA_1c_ values in patients with type 1 diabetes. Furthermore, they showed lower rates of large for gestational age, higher time in range, and fewer adverse neonatal outcomes [46].

In their meta-analysis, Rys et al [47] investigated HbA_1c_ values in pregnant women with type 1 diabetes using continuous subcutaneous insulin infusion or multiple daily injections. They reported lower HbA_1c_ in the first trimester for continuous subcutaneous insulin infusion users (weighted mean difference: −0.45%; 95% CI −0.62 to −0.27).

Research on gestational diabetes–specific mHealth apps is very limited. Skar et al [48] reported gestational diabetes–specific mHealth apps to be effective in increasing the confidence of women with gestational diabetes in their self-management and their motivation for behavior changes. In addition, Chen et al [49] concluded that gestational diabetes specific mHealth apps can provide time- and cost-efficient personalized interventions to improve gestational diabetes management and clinical outcomes. Yu et al [50] reported a clear superiority of continuous glucose monitoring system compared to self-monitoring blood glucose in detecting hypo- and hyperglycemic episodes.

### Conclusions

Technologies for diabetes seem to have a particularly positive effect on glycemic control in all types of diabetes. In pregnant women with type 1 diabetes, continuous glucose monitoring system as well as closed-loop systems seem to improve glycemic control. In women with gestational diabetes, the use of continuous glucose monitoring system systems has been shown, by this review, to improve glycemic control. mHealth apps can also improve glycemic control as well as certain pregnancy and birth related and neonatal outcomes in women with gestational diabetes.

Furthermore, this review showed that there is a lack of research on the clinical effectiveness of technologies for pregnant women with type 2 diabetes. In addition, sample sizes were, in many cases, rather small. Further research is needed to gain more firm evidence on the clinical effectiveness of diabetes technologies in pregnant women.

## Appendix

Multimedia Appendix 1 Search strategies.

Multimedia Appendix 2 PRISMA flow diagram.

Multimedia Appendix 3 Quality assessment using Effective Public Health Practice Project criteria.

## Abbreviations

CG: control group
EPHPP: Effective Public Health Practice Project
HbA_1c_: hemoglobin A_1c_
IG: intervention group
MQ: moderate quality
PRISMA: Preferred Reporting Items for Systematic Reviews and Meta-Analyses
SQ: strong quality
WQ: weak quality
