# Supplementary material for: Clinical Effectiveness of Different Technologies for Diabetes in Pregnancy: Systematic Literature Review
Source: J Med Internet Res. 2021 Apr 28;23(4):e24982. doi: 10.2196/24982 (PMC8116994; doi:10.2196/24982)
Supplement: Multimedia Appendix 1 [file jmir_v23i4e24982_app1.docx]

# **Search strategies.**

|  | PUBMED | EMBASE | COCHRANE | CINAHL | WEB OF SCIENCE  CORE COLLECTION |
| --- | --- | --- | --- | --- | --- |
| **FILTERS** | Publication date 2008/01/01 to 2020/12/31; English; German | Publication date 2008­­­-2020; English; German | Publication date 2008/01/01-2020/09/05; English; German; Trials | Publication date 2008/01/01-2020/12/31; English, German | Publication date 2008-2020; English; German |
| **STRATEGY** | ((("Diabetes Mellitus"[Mesh]) AND ("Pregnancy"[Mesh])) OR ("Diabetes, Gestational"[Mesh])) AND ((((((("Mobile Applications"[Mesh]) OR ("Insulin Infusion Systems"[Mesh])) OR ("insulin pump"[Title/Abstract])) OR ("flash glucose monitoring"[Title/Abstract])) OR (continuous glucose monitoring[Title/Abstract])) OR (CGM[Title/Abstract])) OR (FGM[Title/Abstract])) | (( ‘pregnancy diabetes mellitus’/exp) AND (‘Mobile Applications’/exp OR ‘Insulin Infusion’/exp OR ‘insulin pump’/exp OR ‘flash glucose monitoring’/exp OR ‘continuous glucose monitoring system’/exp OR ‘CGM’:ab,ti OR ‘FGM:ab,ti)) | (((MeSH [Diabetes Mellitus] AND MeSH [Pregnancy]) OR MeSH [Diabetes, Gestational])) AND (MeSH [Insulin Infusion Systems] OR MeSH [Mobile Applications] OR “insulin pump”:ti,ab OR “flash glucose monitoring”:ti,ab OR “continuous glucose monitoring”:ti,ab OR “CGM”:ti,ab OR “FGM”:ti,ab)) | ((MH “diabetes mellitus” AND MH “pregnancy”) AND (MH “mobile applications” OR TI insulin infusion OR AB insulin infusion OR TI continuous glucose monitoring OR AB continuous glucose monitoring OR TI insulin pump OR AB insulin pump OR TI Insulin Infusion OR AB Insulin Infusion OR TI flash glucose monitoring OR AB flash glucose monitoring OR TI CGM OR AB CGM OR TI FGM OR AB FGM)) | (((TOPIC “diabetes mellitus” AND “pregnancy”) OR (TOPIC “gestational diabetes”)) AND (TOPIC mobile applications OR TOPIC insulin infusion OR TOPIC insulin pump OR TOPIC flash glucose monitoring OR TOPIC continuous glucose monitoring OR TOPIC CGM OR TOPIC FGM))) |
