# Supplementary material for: Clinical Effectiveness of Different Technologies for Diabetes in Pregnancy: Systematic Literature Review
Source: J Med Internet Res. 2021 Apr 28;23(4):e24982. doi: 10.2196/24982 (PMC8116994; doi:10.2196/24982)
Supplement: Multimedia Appendix 2 [file jmir_v23i4e24982_app2.docx]

PRISMA flow diagram.

Records screened (titles/abstracts)
(n = 754)

Full-text articles excluded
(n = 38)

- Study design (n=15)
- Retrospective (n=17)
- Study protocol (n=3)
- Type(s) of DM not reported (n=3)

Records excluded based on inclusion and exclusion criteria
(n = 693)

Records identified through database searching in September 2020 (n = 974)

PubMed: n=204

CINAHL: n=20

Cochrane Library: n=207

Web of Science Core Collection: n=196

EMBASE: 347

Additional records identified through other sources (reference lists/ Google Scholar)
(n = 3)

Full-text articles assessed for eligibility
(n = 61)

Studies included in qualitative synthesis
(n = 22)

Records after duplicates removed
(n = 754)

## Identification

## Eligibility

## Included

## Screening
