# Supplementary material for: Clinical Effectiveness of Different Technologies for Diabetes in Pregnancy: Systematic Literature Review
Source: J Med Internet Res. 2021 Apr 28;23(4):e24982. doi: 10.2196/24982 (PMC8116994; doi:10.2196/24982)
Supplement: Multimedia Appendix 3 [file jmir_v23i4e24982_app3.docx]

**Quality assessment using “Effective Public Health Practice Project” (EPHPP).**

| Question/ Study | A Seletion Bias (Q1) | A Selection Bias (Q2) | A SCORE | B Study Design | B SCORE | C  Confoun-ders (Q1) | C  Confoun-ders (Q2) | C SCORE | D Blinding (Q1) | D Blinding (Q2) | D SCORE | E  Data collection (Q1) | E  Data Collection (Q2) | E SCORE | F Withdrawals and Drop-outs (Q1) | F Withdrawals and Drop-outs (Q2) | F SCORE | GLOBAL RATING |
| --- | --- | --- | --- | --- | --- | --- | --- | --- | --- | --- | --- | --- | --- | --- | --- | --- | --- | --- |
| Type 1 Diabetes Mellitus | | | | | | | | | | | | | | | | | |  |
| Cordua et al. (2013) | 1 | 2 | ** | 1 | *** | 2 | / | *** | 3 | 3 | ** | 1 | 1 | *** | 1 | 1 | *** | **Strong ***** |
| Feig et al. (2017) | 1 | 5 | * | 1 | *** | 2 | / | *** | 3 | 3 | ** | 1 | 1 | *** | 1 | 1 | *** | **Moderate **** |
| Petrovski et al. (2011) | 1 | 1 | *** | 1 | *** | 2 | / | *** | 3 | 3 | ** | 1 | 1 | *** | 1 | 1 | *** | **Strong ***** |
| Cyganek et al. (2010) | 2 | 1 | ** | 3 | ** | 2 | / | *** | 3 | 3 | ** | 1 | 1 | *** | 2 | / | *** | **Strong ***** |
| Jotic et al. (2020) | 2 | 1 | ** | 3 | ** | 2 | / | *** | 3 | 3 | ** | 1 | 1 | *** | 1 | 1 | *** | **Strong ***** |
| Feig et al. (2018) | 1 | 5 | * | 1 | *** | 2 | / | *** | 3 | 3 | ** | 1 | 1 | *** | 1 | 1 | *** | **Moderate **** |
| Gutaj et al. (2015) | 1 | 1 | *** | 1 | *** | 2 | / | *** | 3 | 3 | ** | 1 | 1 | *** | 1 | 1 | *** | **Strong ***** |
| Stewart et al. (2016) | 1 | 5 | * | 1 | *** | 2 | / | *** | 3 | 3 | ** | 1 | 1 | *** | 1 | 1 | *** | **Moderate **** |
| Stewart et al. (2018) | 1 | 5 | * | 1 | *** | 2 | / | *** | 3 | 3 | ** | 1 | 1 | *** | 1 | 1 | *** | **Moderate **** |
| Gestational Diabetes Mellitus | | | | | | | | | | | | | | | | | |  |
| Alfadhli et al. (2016) | 4 | 1 | * | 1 | *** | 2 | / | *** | 3 | 3 | ** | 1 | 1 | *** | 1 | 1 | *** | **Moderate **** |
| Paramasivam et al. (2018) | 4 | 2 | * | 1 | *** | 2 | / | *** | 1 | 3 | ** | 1 | 1 | *** | 1 | 2 | ** | **Moderate **** |
| Wei et al. (2016) | 1 | 5 | *** | 1 | *** | 2 | / | *** | 3 | 3 | ** | 1 | 1 | *** | 1 | 1 | *** | **Moderate **** |
| Lane et al. (2019) | 1 | 1 | *** | 1 | *** | 2 | / | *** | 3 | 3 | ** | 1 | 1 | *** | 1 | 1 | *** | **Strong ***** |
| Guo et al. (2019) | 1 | 1 | *** | 1 | *** | 2 | / | *** | 3 | 3 | ** | 1 | 1 | *** | 1 | 1 | *** | **Strong ***** |
| Mackillop et al. (2018) | 1 | 1 | *** | 1 | *** | 3 | 4 | * | 3 | 3 | ** | 1 | 1 | *** | 1 | 1 | *** | **Weak *** |
| Bromuri et al. (2016) | 1 | 5 | *** | 1 | *** | 2 | / | *** | 3 | 3 | ** | 1 | 1 | *** | 1 | 1 | *** | **Moderate **** |
| Yang et al. (2018) | 1 | 5 | *** | 2 | *** | 2 | / | *** | 3 | 3 | ** | 1 | 1 | *** | 1 | 1 | *** | **Moderate **** |
| Miremberg et al. (2018) | 1 | 1 | *** | 1 | *** | 2 | / | *** | 3 | 3 | ** | 1 | 1 | *** | 1 | 1 | *** | **Strong ***** |
| Borgen et al. (2019) | 1 | 1 | *** | 1 | *** | 3 | 4 | * | 3 | 3 | ** | 1 | 1 | *** | 1 | 1 | *** | **Weak *** |
| Type 1 and Type 2 Diabetes Mellitus | | | | | | | | | | | | | | | | | |  |
| Secher et al. (2013) | 1 | 2 | ** | 1 | *** | 2 | / | *** | 1 | 1 | * | 1 | 1 | *** | 1 | 1 | *** | **Moderate **** |
| Murphy et al. (2008) | 1 | 2 | ** | 1 | *** | 2 | / | *** | 1 | 1 | * | 1 | 1 | *** | 1 | 1 | *** | **Moderate **** |
| Kernaghan et al. (2008) | 1 | 5 | ** | 2 | *** | 2 | / | *** | 3 | 3 | ** | 1 | 1 | *** | 1 | 1 | *** | **Strong ***** |

* = weak, ** = moderate, *** = strong, 1-5 = response options according to EPHPP, / = not applicable
